# Shiga Toxin 1a Blunts Shiga Toxin 2a-pathogenic Effects in Blood

**DOI:** 10.1055/a-2805-9903

**Published:** 2026-02-24

**Authors:** Elisa Varrone, Luciano Consagra, Giorgia Rossi, Domenica Carnicelli, Paola Paterini, Barbara Brunetti, Francesca Ricci, Pier Luigi Tazzari, Gianluca Storci, Massimiliano Bonafè, Gianluigi Ardissino, Maurizio Brigotti

**Affiliations:** 1 Department of Medical and Surgical Sciences (DIMEC), University of Bologna, BolognaItaly; 2 Center for Applied Biomedical Research-CRBA, University of Bologna, IRCCS Azienda Ospedaliero-Universitaria di Bologna, BolognaItaly; 3 Department of Veterinary Medical Sciences (DIMEVET), University of Bologna, Ozzano dell'Emilia, BolognaItaly; 4 IRCCS Azienda Ospedaliero-Universitaria di Bologna, BolognaItaly; 5 Center for HUS Control, Prevention and Management, Fondazione IRCCS Ca' Granda Ospedale Maggiore Policlinico, MilanItaly

**Keywords:** hemolytic uremic syndrome, acute renal failure, bacterial toxins, Shiga toxin-producing *Escherichia coli*

## Abstract

**Background:**

Once released into human blood, Shiga toxins (Stx) interact with platelets and leukocytes, stimulating them to form aggregates and to release pathogenic extracellular vesicles (EV) containing Stx. These EV are considered the trigger driving the transition from bloody diarrhea to the life-threatening hemolytic uremic syndrome (HUS) during human infections by Stx-producing *Escherichia coli* (STEC). In children, HUS is characterized by hemolytic anemia, thrombocytopenia, and acute renal failure. The risk of any STEC-infected patient of developing HUS varies significantly depending on the Stx type produced by the bacteria, i.e., it is negligible for Shiga toxin 1 (Stx1), relevant for Shiga toxin 2 (Stx2), and considerably reduced when both toxins are present.

**Material and Methods:**

To mimic what happens in the bloodstream of patients, human blood was challenged with Stx2a, Stx1a, or both toxins, and the formation of leukocyte/platelet aggregates was evaluated by direct-flow cytometric analysis. Pathogenic blood cell-derived EV were then isolated, their number and size determined by nanoparticle tracking analysis, and their proteins characterized by capillary Western blotting.

**Results:**

We found that the presence of Stx1a during Stx2a challenge significantly reduced the formation of pathogenic EV, particularly the large (>300 nm) EV population causing HUS development. Notably, the amount of Stx2a significantly decreased in Stx1a + Stx2a-triggered EV with respect to Stx2a-induced EV.

**Conclusion:**

Our findings suggest that in STEC-infected children the presence of Stx1 in association with Stx2 reduces the risk of developing HUS by lowering the release of Stx2-containing blood cell-derived EV which are considered the main culprits for HUS onset.

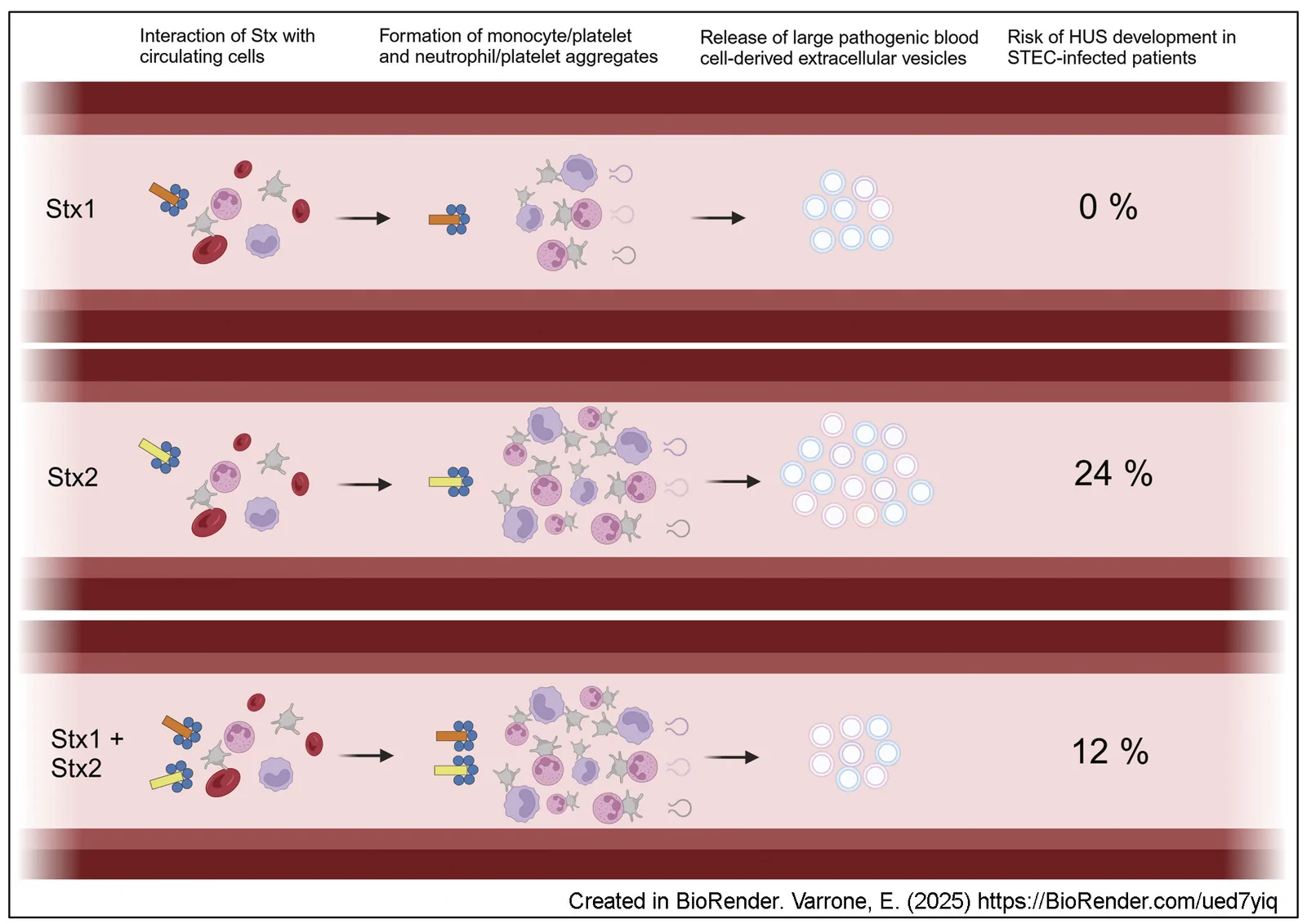

## Introduction

Hemolytic uremic syndrome (HUS) is a thrombotic microangiopathy, presenting with hemolytic anemia, thrombocytopenia, and acute kidney damage, which occurs as a severe life-threatening sequela of Shiga toxin-producing *Escherichia coli* (STEC) intestinal infections. The major virulence factors released by these pathogenic bacteria include powerful exotoxins known as Shiga toxins (Stx). 1 HUS most often manifests in childhood and is considered the leading cause of acute kidney injury in previously healthy children aged <5 years. Approximately 15 to 20% of STEC-infected pediatric patients develop HUS, 6 to 7 days after a prodromal intestinal phase, while in 80 to 85% of cases the gastroenteritis goes into spontaneous resolution. 2 3

STEC are non-invasive microorganisms that enter the human body and specifically colonize the gut. The primary disease manifestation is watery diarrhea, which appears 3 days after the ingestion of contaminated food or water, followed by bloody diarrhea, which usually develops 1 to 3 days after the onset of symptoms. Bloody diarrhea is due to the release of Stx that penetrate the intestinal mucosa and cause lesions to gut microvasculature. 3 4 5 6 Toxins are then transported in the bloodstream causing injuries mainly to the endothelial cells of the main target organs: the kidney and the brain. 1 3

During full-blown HUS, free Stx are rarely found in patients' blood. 7 8 9 10 Conversely, they are found in blood before the onset of HUS, in free form or associated with platelets, neutrophils, monocytes, and erythrocytes. The interaction of Stx with blood cells stimulates them to form aggregates and to release large extracellular vesicles (EV) containing Stx which are considered responsible for the transition from bloody diarrhea to HUS. 1 7 8 11 12 13 14 The formation of leukocyte/platelet aggregates is mainly related to the exposition of P-selectin on the membrane of activated platelets and to its interaction with the constitutively expressed P-selectin glycoprotein ligand found on leukocytes. 15 16 Large EV responsible for toxin delivery to target endothelial cells are readily released by circulating cells that are resistant to toxin action and become activated after interacting with Stx through different receptors. Contrariwise, target cells sensitive to toxin action were found to release small Stx2-containing EV 1 that may contribute to local toxin spreading rather than to toxin delivery in blood, as may occur early in intestinal endothelia (bloody diarrhea) or later in renal and cerebral endothelia (overt HUS).

Stx are a family of AB5 bacterial toxins consisting of two main types, Stx1 and Stx2, each with several subtypes (4 for Stx1, and 12 for Stx2) with different receptor preference and toxic potency. Stx2a is the subtype mainly involved in the development of HUS in humans. 17 Stx are composed of a single A chain, which contains the enzymatic domain, non-covalently bound to five identical B chains. 18 Upon binding to the functional receptor globotriaosylceramide (Gb3Cer) and, to a lesser extent, to globotetraosylceramide (Gb4Cer) through the B pentameric ring, 19 20 the toxins are endocytosed and undergo retrograde transport to the cytoplasm where they express the deadenylating activity on 28S rRNA in ribosomes, reducing ribosomal affinity for eukaryotic elongation factors (eEF1 and eEF2) and leading to irreversible arrest of translation. 1

Since Stx1 and Stx2 are encoded by bacteriophages, STEC strains carrying more than one bacteriophage can produce more than one toxin type. 4 21 Epidemiological studies indicate that strains which produce only Stx1 are not usually associated with HUS, 22 or are associated with milder symptoms. 23 24 In addition, it has been reported that HUS is more commonly associated with STEC strains expressing Stx2 than with strains carrying Stx1 and Stx2 together. 17 25 These findings were confirmed by data from a 10-year bloody diarrhea screening program for STEC infection in humans (<20 years) involving 63 pediatric units in Northern Italy (ItalKid-HUS Network) and 2.3 million pediatric referral population. 26 The study revealed that in Stx-positive infections the risk of progressing to HUS depends on toxin type produced by the bacteria: 0% for Stx1, 23.7% for Stx2, and 12.7% for Stx1 + Stx2. 26 These findings suggest that the presence of Stx1 in combination with Stx2 reduces the pathogenetic potential of Stx2 itself. 26 27 However, the mechanism underlying these epidemiological observations needs to be further clarified. Although Stx1a and Stx2a have nearly 60% amino acid homology, 23 their enzymatic activity and the molecular mechanism are identical. 4 The main difference between the two subtypes is in their receptor binding sites in the B subunits, resulting in a different affinity for the specific receptor. 28 29

Experiments conducted using a HUS animal model obtained by infecting mice with a STEC strain isolated from a patient with bloody diarrhea that produced both Stx1 and Stx2 (O26:H11 strain TW08571) showed that the administration of a Stx2-neutralizing antibody, but not Stx1-neutralizing antibody, protected mice from infection, suggesting that Stx2a was responsible for the toxic effects. 24 Surprisingly, the same study showed that Stx1 even decreased the toxicity of Stx2. Mice orally co-intoxicated with Stx2a together with various concentrations of Stx1a showed improved dose-dependent survival rates and a longer mean time to death compared with animals intoxicated with Stx2a only. Furthermore, following co-intoxication, serum creatinine and sodium levels turned to be normal while these renal parameters were pathologic in animal treated with Stx2a alone. 30

Further evidence of Stx1a-induced protection in the presence of Stx2a was obtained by bypassing the gastrointestinal tract by the systemic intoxication of mice with Stx2a with or without the Stx1a B subunit (50 ng) given several hours before the Stx2a challenge. 24 In this experimental setting Stx1a B subunit reduced the toxic effects of Stx2a. A competition effect between the two toxins at kidney level was proposed by the authors. However, the two toxins bind to the same renal regions of the kidney and a decreased binding of Stx2a in the presence of Stx1a was not observed. 24

To conclude, the different co-intoxication mouse models replicated the reduced severity of symptoms associated with human infections with STEC strains positive for both toxins as opposed to those positive for Stx2a alone.

Here we investigated whether co-intoxication of human blood with Stx1a and Stx2a reduces the formation of leukocyte/platelet aggregates and the release of pathogenic blood cells-derived EV compared with the treatment with Stx2a or Stx1a alone.

## Methods

### Shiga Toxins

Stx2a and Stx1a were produced from *E. coli* C600–933W and from *E. coli* C600-H19J, respectively (kindly donated by Dr. Alison O'Brien; Department of Microbiology and Immunology, Uniformed Services University of the Health Sciences, Bethesda, MD, USA). Stx were purified by receptor analogue affinity chromatography with (Stx1a) globotriose–Fractogel (IsoSep, AB) 31 or (Stx2a) cyanogen bromide-activated Sepharose coupled to bovine serum albumin (BSA) linked to a terminal galabiose (Galα1–4Galβ- *O* -spacer—BSA, Glycorex, Lund, Sweden). 32 The isolated toxins were then applied to an ActiClean Etox column (Sterogene Bioseparations, Carlsbad, CA, USA) to reduce the endotoxin contamination. The purified toxins were quantified by a Lowry assay and tested using Limulus Pyrogen Plus amebocyte lysate (Cambrex, Walkersville, MD, USA), showing the presence of a low amount of contaminating lipopolysaccharide (LPS) (8.1 ng/mg Stx1a and 6.5 ng/mg Stx2a). Toxins were stored at −80°C in small aliquots and diluted with phosphate buffered saline (PBS) containing 1% BSA to avoid nonspecific binding to plastic tubes.

### Blood Collection from Healthy Donors

After obtaining the informed consent, blood samples from six healthy adult volunteers who were recruited at the Emilia-Romagna Regional Blood Centre (Maggiore Hospital, AUSL Bologna, Italy) were used in the study. The median age of the donors was 57.5 years (range: 40–66) and the AB0/Rh groups were A+ (3), 0− (2), and 0+ (1). Blood samples were anticoagulated with CPD (citrate-phosphate-dextrose solution).

### Detection of Leukocyte/Platelet Aggregates

Direct fluorescence-based flow cytometry was used to assess the generation of leukocyte/platelet aggregates. Non-fractionated blood samples (1 mL) were incubated with Stx1a (1–2 nM), with Stx2a (1–2 nM), with different combinations of both toxins, or with vehicles for 4 hours at 37°C. After the osmotic lysis of the erythrocytes, the samples were incubated with different fluorescent monoclonal antibodies (mAb). These included anti-CD41 labeled with phycoerythrin (PE) to detect platelets, anti-CD14 labeled with FITC to detect monocytes, and anti-CD16 labeled with phycoerythrin cyanine 5 (PC5) to detect neutrophils. To further eliminate false positives, controls were performed using the proper isotopic antibodies. Monocyte/platelet aggregates were identified as the cell populations exhibiting double CD14/CD41 positivity, and neutrophil/platelet aggregates as the granular cell populations exhibiting double CD16/CD41 positivity.

### Isolation of Blood Cell-derived Extracellular Vesicles

After being diluted with an equal volume of Dulbecco's Modified Eagle Medium (DMEM) supplemented with the fibrin polymerization inhibitor Gly-Pro-Arg-Pro (GPRP, 10 µM; Merck), 40 mL of whole blood were incubated for 4 hours at 37°C with 1 nM Stx1a or 2 nM Stx2a, or 2 nM Stx2a + 1 nM Stx1a or vehicle. At the end of incubation, EV were isolated by differential centrifugation: 2,600 g for 35 minutes to discard platelets and blood cells; 10,400 g for 25 minutes to remove cell debris and apoptotic bodies; and 20,800 g for 1 hour to obtain large EV (10,400–20,800 g). After resuspending the EV in 500 µL of PBS, a 250 µL aliquot was centrifuged at 20,800 g for 40 minutes, and the pellet was frozen at –80°C to prepare vesicle-derived proteins for additional characterization. The remaining 250 µL aliquot was centrifuged as described above, and the pellet was then resuspended in 34 µL of PBS for the determination of the EV number and size. For gel-filtration analysis the whole EV preparation was not divided into aliquots and, after centrifugation, the pellet was resuspended in 100 µL of PBS.

### Analysis of Extracellular Vesicles by Gel Filtration

Exclusion size chromatography was performed with qEV original 70 nm columns (Izon, New Zealand) according to the manufacturer's instructions. Samples (100 µL brought to 500 µL with PBS) of EV preparations from human blood treated with Stx1a (1 nM), Stx2a (2 nM), or PBS (see above) were applied to the columns equilibrated with PBS; then 500 µL fractions were obtained by passing the same buffer. The elution profile of the fractionated EV was obtained by measuring the toxicity for Vero cells of aliquots of the different fractions.

### Vero Cells Viability Assay

Vero cells (50,000 in 500 µL of medium) were seeded in 24-well plates and cultured in DMEM supplemented with 10% FBS, 2 mM L-glutamine and 1% penicillin-streptomycin in incubators at 37°C in a water-saturated 5% CO _2_ atmosphere in air. After 24 hours, cells were treated for 72 hours with aliquots of the fractions obtained by the analytical gel filtration described above. According to the well-known different sensitivity of Vero cells to the two Stx type (Stx1a > Stx2a), 24 1 or 100 μL of the fractions were used for Stx1a EV or Stx2a EV chromatography, respectively. In the case of control EV, 100 μL of the fractions were applied to the column. The percentage of live cells at the end of incubation was measured by a luminescent cell viability assay (Cell titer-Glo, Promega, Milan, Italy) by quantifying the amount of ATP as a product of live cells. After removal of the incubation medium, 150 µL of fresh medium was added and the sample further supplemented with an equal volume of Cell Titer-Glo Reagent. After shaking for 2 minutes to allow cell lysis, 200 µL aliquots of the mixture were transferred to a 96-well plate and incubated for 10 minutes at room temperature. The amount of ATP in the samples was measured according to the manufacturer's instructions by recording a luminescent signal proportional to the amount of the nucleotide.

### Analysis of Extracellular Vesicles by Nanoparticle Tracking Analysis (NTA)

Nanoparticle tracking analysis (NTA) with the NanoSight RS300 system (Malvern Panalytical, Malvern, United Kingdom) was used to measure the size and relative abundance of the isolated EV. PBS filtered at 0.1 μm was used to dilute each sample (1:1,000). To determine the average number of dimensions and concentration (particles/mL), three videos, each lasting 60 seconds, were captured and examined with NTA 3.2 analytical software. Instrument characteristics and setting: camera, NSCMOS; laser, 532 nm; syringe pump, flow rate 30 (a.i.); screen gain, 1; camera level, 15–16; detection threshold, 3–4; particle/frame during acquisition, above 18–20 particle/frame.

### Protein Extraction from Extracellular Vesicles

Each pellet (see above) was lysed by adding 25 µL of RIPA buffer containing a complete protease inhibitor cocktail (Roche, Sigma-Aldrich, USA). The incubation period was 10 minutes, with occasional shaking. At the end of incubation, debris were removed by centrifuging at 14,000 g for 10 minutes at 4°C. The supernatant was then collected and frozen at −80°C. Using BSA as the standard, EV-derived proteins were quantified with the Bradford assay.

### Characterization of Blood Cell-derived Extracellular Vesicles by Capillary Western Blot

Quantitative capillary Western Blot (WES) was used to analyze EV-derived proteins. According to the manufacturer's instructions, the proteins were diluted with a master mix containing DTT and incubated for 5 minutes at 95°C. Subsequently, each well of the plate was filled with the marker, sample, primary and secondary antibodies, antibody diluent for the blocking step, streptavidin-HRP, and luminol–peroxide mix. The automated Western Blot analysis and quantitative immunodetection (Protein Simple Western, Biotechne) were initiated using the plate and capillaries. Different antibodies were employed: rabbit polyclonal anti-Alix 1:50 (Novusbio) as extracellular vesicle marker; rabbit polyclonal anti-Stx2a 1:50 (Dr. Stefano Morabito, ISS Rome) to detect Stx2a; mouse monoclonal anti-CD45 1:250 (BD Transduction Laboratories) as leukocyte marker; rabbit polyclonal anti-CD42a 1:10 (GeneTex) as platelet marker.

### Statistics

Statistical analysis was performed using GraphPad Prism 8 software. Differences in continuous variables (means, SD) were tested with the unpaired *t* -test after assessing the normality of their distribution. A *p* -value <0.05 was considered statistically significant.

## Results

We sought to assess in vitro the effect of the presence of Stx1a in addition to Stx2a on the early toxaemic phenomena that occur before the onset of HUS in patients, i.e., the formation of leukocyte/platelet aggregates and the release of large pathogenic EV by circulating cells.

Nanomolar concentrations of the two toxins were used to challenge human blood and allow toxin binding to circulating cells, according to earlier determinations performed in patients' blood. 1 33

### Effect of Stx1a, Stx2a, and Their Combination on the Formation of Leukocytes/Platelets Aggregates

Human blood samples from three different donors were treated with nanomolar concentrations of both toxins, either alone or in combination, for 4 hours at 37°C to allow the formation of neutrophil/platelet (CD16 +/CD41 + ) or monocyte/platelet (CD14 +/CD41 + ) aggregates detected by flow cytometry ( Fig. 1 ) and shown in the blood smears stained with May-Grünwald Giemsa ( Supplementary Fig. S1 , available in the online version only). Histologic examination revealed that fibrin was virtually absent, confirming that the formation of the above-mentioned aggregates precedes the development of thrombi in renal microvasculature (Stx2a-triggered aggregate in Supplementary Fig. S1 , available in the online version only). The cytofluorimetric results expressed as the percentage of aggregates on the total neutrophil and monocyte population are shown in Fig. 1 . The two toxins behaved differently as Stx2a stimulated the production of a greater number of monocyte/platelet and neutrophil/platelet aggregates compared with Stx1a, especially at 2 nM concentration. Moreover, dose-dependent effects were observed only with Stx2a. This is confirmed by comparing the extent of aggregates formed at 2 nM Stx2a (approximately 80% of leukocyte populations) with that recorded when the 2 nM toxin concentration was obtained by simultaneously adding 1 nM of both Stx1a and Stx2a (approximately 40% of leukocyte populations) ( Fig. 1A, B ).

**Fig. 1:**
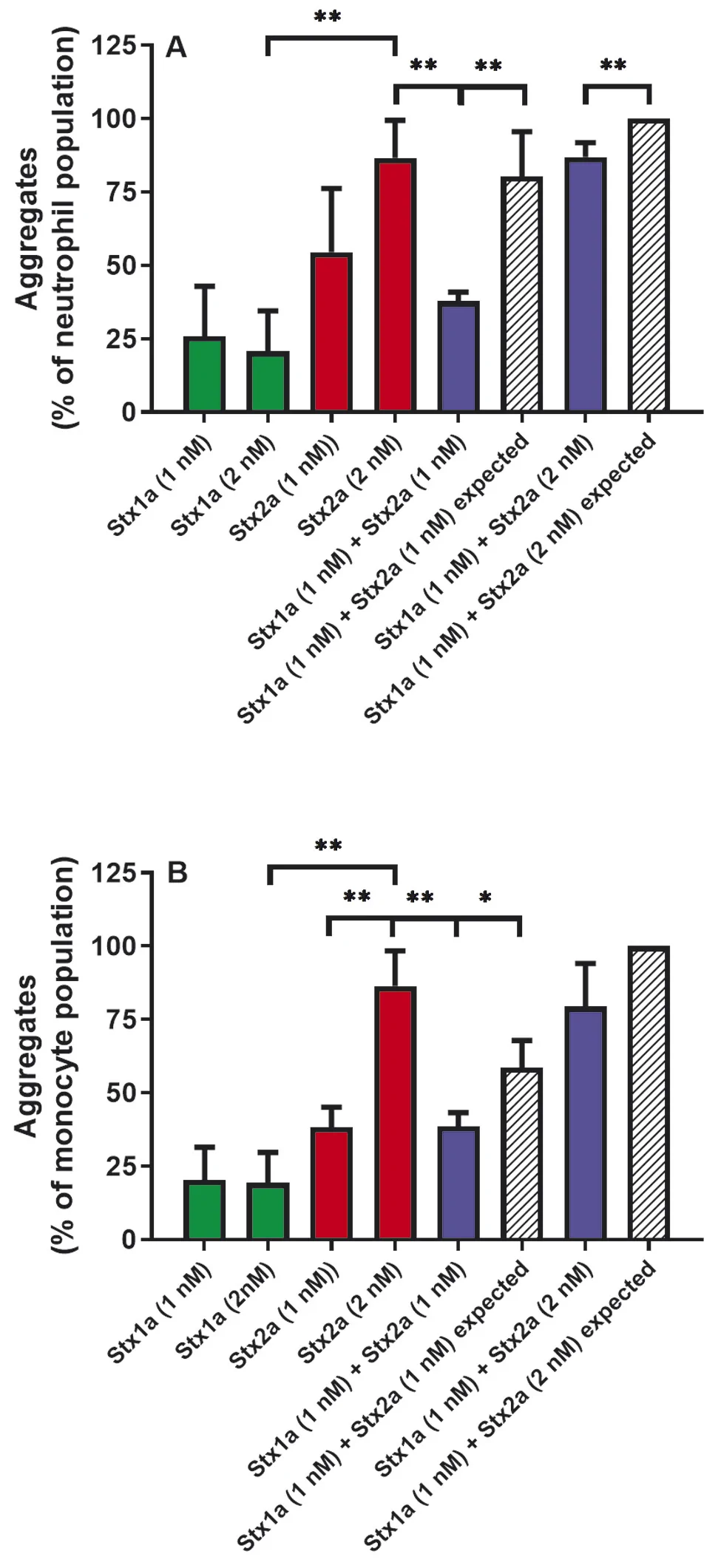
Effect of Stx1a, Stx2a and their combination on the formation of leukocytes/platelets aggregates. Human blood samples (1 mL treated for 4 hours at 37°C with nanomolar concentrations of Stx2a, Stx1a or both were used to compare the effects of toxins on the formation of neutrophil/platelet (CD16+/CD41+) ( **A** ) or monocyte/platelet (CD14+/CD41+) ( **B** ) aggregates by direct flow cytometric analysis. Values (means ± SD) are expressed as percentage of total aggregates on the whole population of neutrophils or monocytes from three different donors after subtraction of the values obtained in each experiment in the absence of toxins (control values). The mean control values ± SD were 3.3% ± 2.7 and 2.3% ± 1.1 for neutrophils and monocytes, respectively. *p < 0.05; **p < 0.01.

Stx1a (1 nM) added to Stx2a (1 nM or 2 nM) did not significantly reduce the formation of aggregates as emerged from the comparison of the red and blue histograms in Fig. 1A, B . Moreover, the lack of additive effects after the combined treatment is evident since the simultaneous addition of 2 nM Stx2a + 1 nM Stx1a induced the formation of lower percentages of aggregates than expected, i.e., than the sum of the values obtained when each toxin was separately added to human blood ( Fig. 1A, B , dotted bars). This phenomenon was even more pronounced with the combination of the two toxins at 1 nM concentration. To sum up, with regards to the formation of leukocyte/platelet aggregates, the presence of Stx1a did not worsen or prevent the pathogenic effects induced by Stx2a.

### Stx1a and Stx2a are Similarly Associated with Large Blood Cell-derived Extracellular Vesicles

To mimic the other phase of the pathogenesis, whole blood from a representative donor was challenged with Stx1a (1 nM) or Stx2a (2 nM) or vehicle, and then large (10,400–20,800 g) blood cell derived-EV were isolated by differential centrifugation and analyzed by gel filtration (qEV size exclusion chromatography). Aliquots of the different fractions were added to Stx-sensitive cells (Vero cells) and their effects measured by a viability assay. Lethal effects were negligible when control EV fractions were assayed as shown by the flattened curve in Fig. 2 , whereas in the case of treatment with Stx1a or Stx2a EV, the toxic activity peaked at fraction 6 ( Fig. 2 ). According to the performance characteristics of qEV original 70 nm columns, when human plasma is applied to the column, total non-purified EV gave a more delayed elution profile (fractions 7–10, blue rectangle in Fig. 2 ). This confirms that both toxins stimulated circulating cells to release large EV and that Stx1a and Stx2a were similarly associated with the largest EV components, as previously demonstrated for Stx2a EV by employing a similar technique. 33 Cytotoxicity assays are obviously not useful for studying the effect of the simultaneous addition of both Stx to human blood, since the toxic effects induced by Stx1a or Stx2a associated with EV are indistinguishable.

**Fig. 2:**
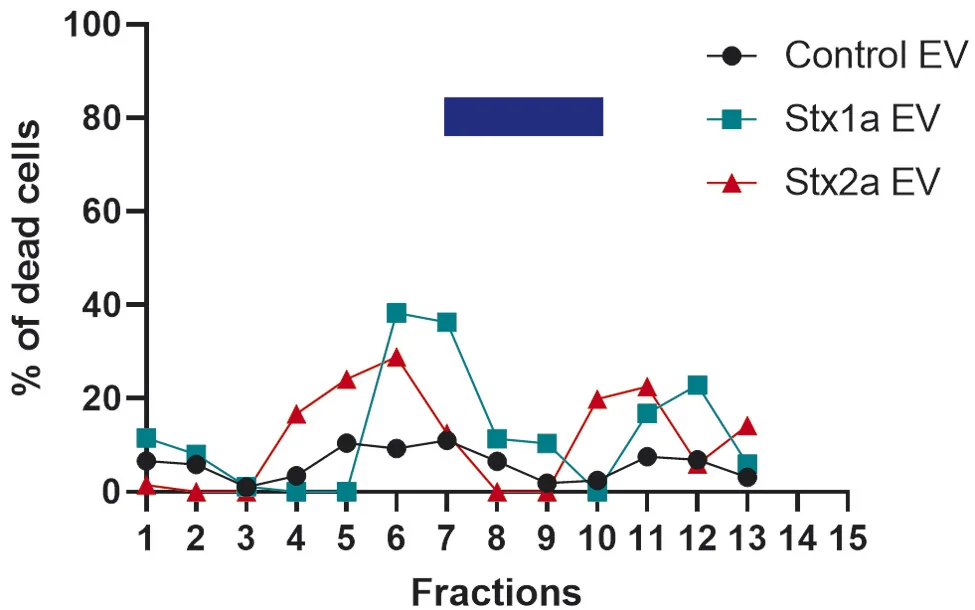
Analytical gel-filtration of extracellular vesicles produced after treatment of human blood with Stx1a or Stx2a. Human blood samples (40 mL) from a representative donor were incubated for 4 h at 37°C with Stx2a (2 nM) or Stx1a (1 nM) or PBS. Large EV were sedimented between 10,400 g and 20,800 g and 100 μL-aliquots of each preparation, brought to 500 μL with PBS, were applied to qEV original 70 nm columns and analysed by size exclusion chromatography as described in Materials and Methods. Aliquots of each 500 μL-fraction were added to cultured Vero cells which are sensitive to Stx action. Viability of cells was assessed by a luminometric assay as described in Material and Methods. Data are expressed as percent of dead cells with respect to controls run with PBS; 1 nM Stx1a EV (green line), 2 nM Stx2a EV (red line), and control EV (black line). According to the column performance described by the manufacturer, the blue rectangle indicates the elution position of total non-purified EV present in human plasma samples (500 μL) loaded on the same column and analysed under the same conditions.

### Stx1a Reduced the Release of Blood Cell-derived Extracellular Vesicles Containing Stx2a

Whole blood from three healthy donors was incubated as described above with Stx2a (2 nM) or Stx1a (1 nM) or with the combination of the two toxins, and then the large (10,400–20,800 g) blood cell-derived EV involved in the pathogenesis of HUS were isolated. For the combined treatment, we chose to closely mimic the situation observed in STEC-infected patients in which, in the presence of both toxins, the blood concentration of Stx2 was not lower than that found in patients presenting only Stx2, 12 26 i.e., Stx1a is present in addition to a fixed higher concentration of Stx2a. It should be noted that under this condition, no effect on the formation of leukocyte/platelet aggregates was observed (see above).

Based on the size-dependent Brownian movements of EV in solution, NTA was performed on the population of EV obtained from a representative donor. In this donor, Stx2a stimulated the production of a greater number (8.5 × 10 ^10^ /mL) of EV ( Fig. 3A , red line) compared with Stx1a (3.3 × 10 ^10^ /mL) ( Fig. 3A , green line) or controls (3.7 × 10 ^10^ /mL) ( Fig. 3A , black line). The control samples showed main peaks with a diameter between 50 and 350 nm ( Fig. 3A ), and two very minor peaks ranging around 480 and 575 nm ( Fig. 3B , black line). The pattern of the smaller EV was very similar in the presence of Stx1a (peaks ranging between 50 and 350 nm diameter) ( Fig. 3A , green line), while a new peak of larger EV, around 400 nm diameter, emerged ( Fig. 3B , green line, black arrow). A similar peak (approximately at 425 nm) was stimulated in the presence of Stx2a ( Fig. 3B , red line, black arrow), and the peaks corresponding to vesicles between 50 and 350 nm diameter raised up ( Fig. 3A , red line). Surprisingly, the concomitant presence of Stx1a and Stx2a reduced the formation of the total number of EV (2.1 × 10 ^10^ /mL) (blue line), by decreasing the lower size components ( Fig. 3A , blue line) and especially the largest (>300 nm) EV population with the complete disappearance of the population at approximately 400 nm diameter ( Fig. 3B , blue line). A clear-cut picture of the observed differences emerged when we calculated the total volume of the different vesicle populations according to their diameter ( Fig. 4A–D ). In particular, in toxin-stimulated samples the peak featuring the larger EV component (approximately 400 nm diameter) accounted for 20% (Stx1a) or 11% (Stx2a) of the total mass of EV released by blood cells. The concomitant presence of Stx1a and Stx2a strongly reduced the total EV mass ( Fig. 4D ), especially the mass of the larger EV (4%). Notably, in the different donors the total extent of EV stimulation induced by the two toxins, when added separately, showed wide variability, particularly when considering the smaller EV populations (<300 nm diameter). Conversely, a constant finding was the toxin-induced increase of large EV populations (>300 nm diameters) that was blunted in the presence of both toxins ( Fig. 5 ).

**Fig. 3:**
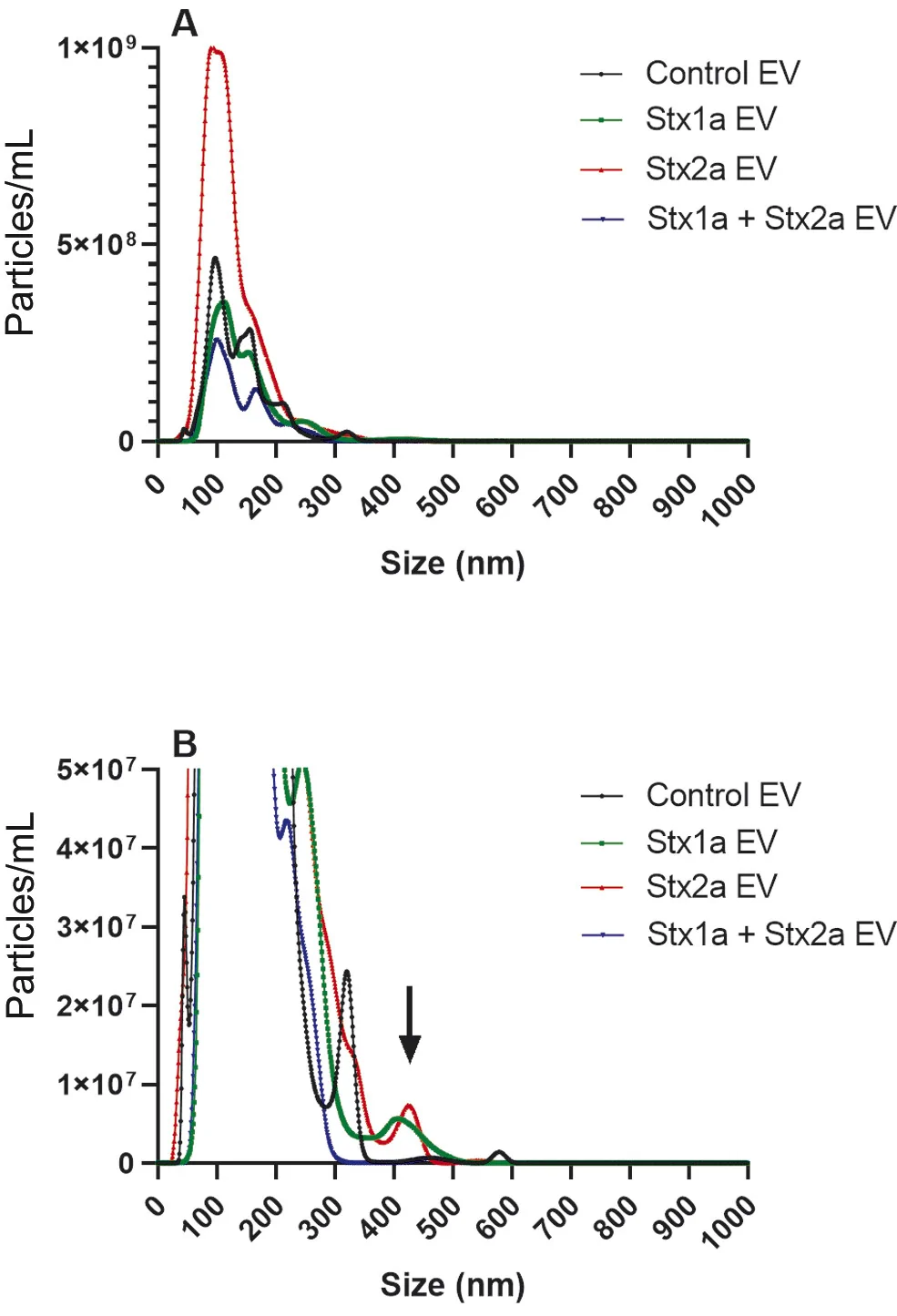
Number of blood cell-derived extracellular vesicles produced after treatment with Stx1a, Stx2a alone or in association. Human blood samples (40 mL) from a representative donor were incubated for 4 h at 37°C with Stx2a (2 nM) or Stx1a (1 nM) or with both toxins (2 nM Stx2a+1 nM Stx1a). Large EV were sedimented between 10,400 g and 20,800 g as described in Materials and Methods and characterized by Nanoparticle tracking analysis. **A** ) Data are expressed as the number of particles/ml of the different EV populations resolved by the technique and differing each other in diameter by 0.5 nm; 1 nM Stx1a (green line), 2 nM Stx2a (red line), 1 nM Stx1a+2 nM Stx2a (blue line) and vehicle (black line). **B** ) The same data depicted in panel A are reported in this panel which differs only in the scale of the y-axis. The black arrow indicates the toxin-induced peaks featuring the larger EV components (~400 nm diameter).

**Fig. 4:**
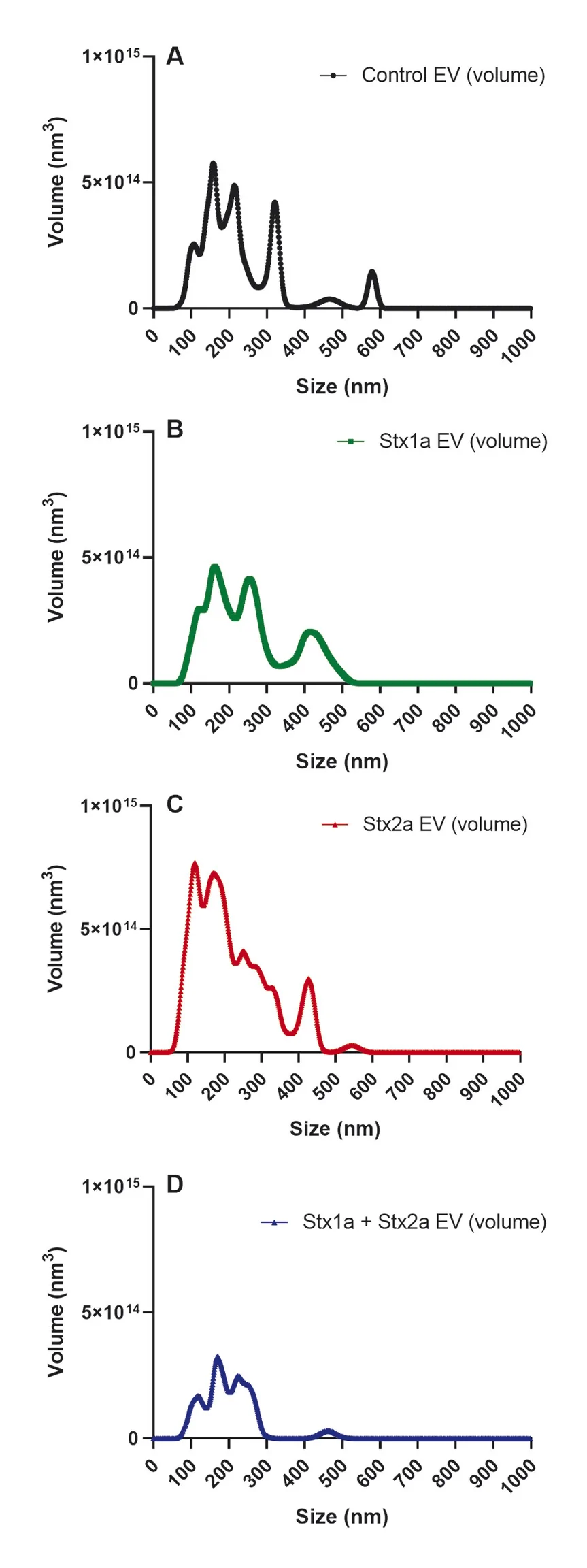
The same data reported in Figure 3 on the large EV released by human circulating cells from a representative donor after treatment with 1 nM Stx1a (green line), 2 nM Stx2a (red line), 1 nM Stx1a+2 nM Stx2a (blue line) and vehicle (black line) were expressed as total volume (nm ^3^ ) of each population of EV and was calculated according to their diameter assuming a spherical shape.

**Fig. 5:**
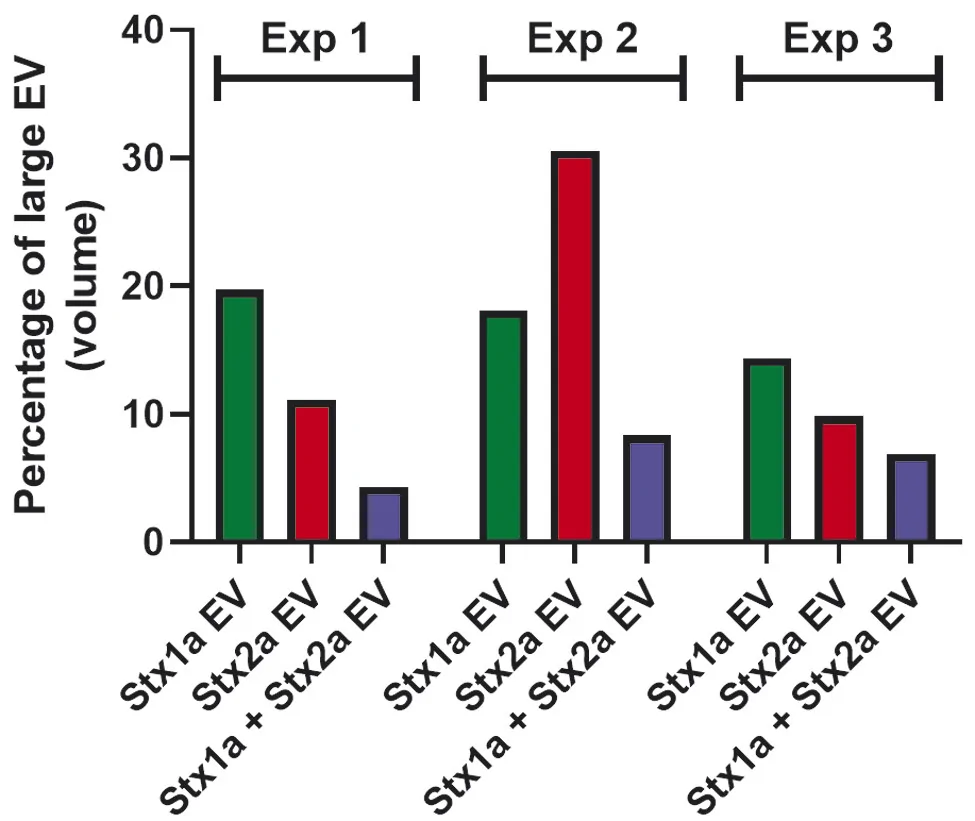
Percentage of large (>300 nm) blood cell-derived extracellular vesicles (volume) produced after treatment with Stx1a, Stx2a alone or in association. Human blood samples (40 mL) from three donors were incubated for 4 h at 37°C with Stx2a (2 nM) or Stx1a (1 nM) or with both toxins (2 nM Stx2a+1 nM Stx1a). Large EV were sedimented between 10,400 g and 20,800 g, as described in Materials and Methods, and characterized by Nanoparticle tracking analysis. Data are expressed as percentage of the volume of large (>300 nm) EV relative to total EV volume calculated with three different donors, 1 nM Stx1a (green bars), 2 nM Stx2a (red bars), 1 nM Stx1a+2 nM Stx2a (blue bars).

Analysis by quantitative capillary Western Blot (WES) of the proteins extracted from the EV' preparations from the three donors confirmed the wide individual variability in the extent of the toxin-induced EV stimulation measured by the expression of the vesicular marker Alix (see SDs in Fig. 6C ). Despite this, a greater expression of Alix was measured in Stx2a-induced EV compared with control EV; the stimulation induced by Stx1a alone was similar whereas that induced by the combined treatment was lower ( Fig. 6A, C ). The slight non-significant decrease in total Alix expression recorded in the latter condition (combined Stx1 + Stx2 treatment in Fig. 6C ) is the result of the focused constant inhibition of the larger EV peaks, which account only for a fraction (10–30%) of the total vesicles ( Fig. 5 ). Again, additive effects were not observed since Alix expression in EV deriving from blood samples treated with both toxins was lower than expected, i.e., lower than the sum of the values obtained when the two toxins were separately added to human blood ( Fig. 6C , dotted bar).

**Fig. 6:**
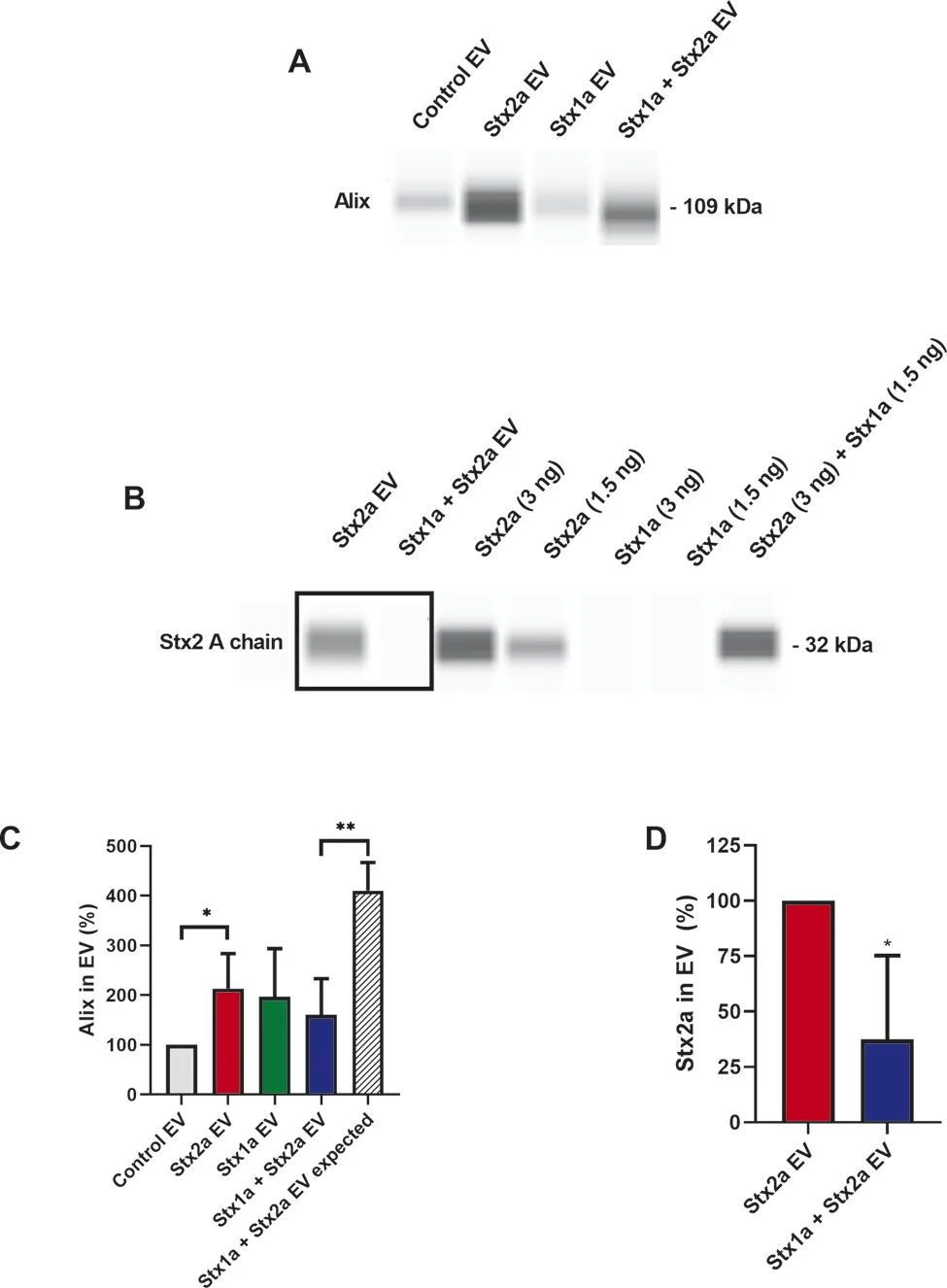
Stx1a reduced the release of blood cell-derived EV containing Stx2. **A** ) WES analysis of the EV marker Alix derived from human EV isolated from a representative donor. **B** ) Quantitative WES analysis of the Stx2a associated to human EV, and ability of the rabbit anti-Stx2 antibody to recognize Stx2a even in the presence of Stx1a. Box: A contrast modification was applied to the lanes with EV-extracted proteins since the amount of Stx2a in these samples was lower (<1.5 ng) than those of the lanes with added Stx. **C** ) Data are expressed as mean ± SD (n=3) of the percentage of Alix determinations with respect to control obtained by analysing the EV isolated from 3 different donors. The expression of Alix in Stx1a+Stx2a-induced EV was also compared to the sum of the values obtained in Stx1a-induced EV and Stx2a-induced EV. **D** ) Data are expressed as mean ± SD (n=3) of the percentage of Stx2a determinations in Stx1a+Stx2a-induced EV relative to Stx2a amounts found in Stx2a-induced EV from three different donors. * *p* < 0.05; ** *p* < 0.01.

As previously demonstrated with Stx2a EV, 33 EV isolated after toxin challenge were positive for the leukocyte marker CD45+ or for the platelet antigen CD42a ( Supplementary Fig. S2 , available in the online version only). The results obtained with the platelet marker paralleled those obtained with the EV marker Alix, as a non-significant decrease in expression was found after the combined treatment, and no additive effects were observed ( Supplementary Fig. S2A, C , available in the online version only). CD45 expression was similar under the different conditions (treatment with Stx1a, Stx2a, or both toxins), although significant stimulation with respect to control EV was observed only in the case of Stx2a ( Supplementary Fig. S2A, B , available in the online version only). Again, no additive effects were induced by the combined treatment.

It is worth noting that the amount of Stx2a associated with EV significantly decreased in Stx1a + Stx2a-induced EV compared with Stx2a-induced EV ( Fig. 6B, D ). The specificity of the anti-Stx2 antibody in recognizing Stx2a by WES, even in the presence of Stx1a, was assessed experimentally. Stx1a (1.5 and 3 ng), Stx2a (1.5 and 3 ng), and a combination of the two toxins (3 ng Stx2a + 1.5 ng Stx1a) were added to proteins extracted from a HeLa cell lysate and WES analysis was performed ( Fig. 6B ). The results clearly showed that the anti-Stx2 antibody was able to recognize Stx2a and not Stx1a, and that the simultaneous presence of Stx1a did not alter the quantitative detection of Stx2a in the samples ( Fig. 6B ). This control experiment confirms that the decreased amount of Stx2a observed in Stx1a + Stx2a-induced EV is not due to artifacts. A further control was performed by comparing control EV and Stx2a-induced EV with the apoptotic bodies (2,600–10,400 g) discarded during EV isolation, showing that the marker Alix and Stx2a were detectable only in 10,400 to 20,800 g EV ( Supplementary Fig. S3 , available in the online version only).

In conclusion, our findings suggest that the presence of Stx1a reduces the amount, size, and Stx2a content of the pathogenic EV triggered by Stx2a upon challenge of human circulating cells. Since Stx2a is more dangerous than Stx1a for STEC-infected patients, the observations herein described might explain why patients infected by STEC strains producing both toxins have lower risks to develop HUS.

## Discussion

It is well known that during the natural course of STEC infections the risk of developing HUS is reduced when bacteria produce both Stx1 and Stx2, being maximal in the presence of STEC producing only Stx2 and minimal when STEC produce Stx1 alone. This epidemiological observation prompted several studies aimed at comparing the toxic effects of these toxins in cellular and animal models.

In cellular models, Stx1a was shown to have greater potency (approximately 10-fold) against Vero cells compared with Stx2a, most likely due to its higher affinity for the target receptor Gb3Cer that allows the toxins to entry the cells. 24 Conversely, the A1 fragment of Stx2, which is responsible for the toxic effect within cells, was found to have higher affinity for ribosomes, hence inhibiting translation in human cells at a significantly higher level. 4 However, these opposite behaviors of the two toxins are not useful for understanding the epidemiological data.

In animal models, it has been demonstrated that Stx2a exhibits greater potency and severity of tissue damage than Stx1a. Differences in toxicity were found when the toxins were parenterally administered, as the lethal dose 50% (LD _50_ ) of Stx2a in mice is 100 times lower than that of Stx1a. 24 In addition, Stx1a is less toxic than Stx2a when given orally to mice; indeed, the LD _50_ of Stx2a was found to be 2.8 µg/mouse, whereas mice intoxicated with Stx1a did not show any signs of morbidity or mortality, even at doses as high as 157 µg/mouse. 30 A possible explanation is that Stx1a might have a reduced capacity to cross the intestinal barrier as compared with Stx2a; however, this hypothesis has been disproved as labeled Stx1 or Stx2 showed similar dissemination patterns and equal accumulation in the kidneys. 30 When the two toxin subtypes were orally co-administered, a protective effect induced by Stx1a on Stx2a toxicity was achieved. This effect was confirmed in the presence of a Stx1a active-site toxoid (with normal B chains and defective A subunit) suggesting that Stx1a reduced Stx2a toxicity by preventing its binding to Gb3Cer receptor expressed by target endothelial cells in the kidney.

In the present paper, we have demonstrated that Stx1a blunts the triggering effect of Stx2a on human circulating cells, thereby reducing the number, the dimension, and the toxic cargo (Stx2a) of the pathogenic EV released by blood cells. It is worth noting that not only Stx2a is clearly associated with HUS development, but that the most ominous form of the toxin is that bound to large EV (100–1000 nm diameters), also characterized by transmission electron microscopy. 12 14 Indeed, the EV populations deriving from blood stimulation by Stx and having diameters larger than 300 nm completely disappeared after the combined treatment. Based on the findings described in previous studies and in the present paper, the most accredited hypothesis is that, in the setting of Stx1 + Stx2-producing STEC infections, Stx1 might exert a protective role via receptor agonism, by occupying the binding site which becomes no longer available for Stx2. This might occur not only during the intoxication of target cells through Gb3Cer receptor, but also during early toxemia when Stx binds to circulating cells through Gb3Cer (monocytes and platelets) and TLR4 (neutrophils, monocytes, and platelets) interfering with the formation and release of EV containing Stx2. The higher affinity of Stx1 for Gb3Cer could be detrimental to the mechanism of EV formation, which relies on the stimulation of both receptors expressed on circulating cells. A possible consequence of such an imbalance might be a decreased release of the harmful Stx2a-containing EV and eventually a milder clinical presentation in patients. Finally, since the dampening effect of Stx1a on the release of Stx2a EV by circulating cells is more marked than that obtained on the formation leukocyte/platelet aggregates, we suggest that aggregate formation is not an absolute prerequisite for EV release, which, on the contrary, strictly depends on the binding to circulating cells.

## Notes

**What is known about this topic?**

- Intestinal infections by *Escherichia coli* that produce Shiga toxins (STEC) can trigger hemolytic uremic syndrome (HUS), the main cause of acute renal failure in early childhood.
- In most cases of STEC-HUS, blood cells are challenged by Shiga toxin 2 (Stx2) and release large pathogenic extracellular vesicles responsible for the development of the syndrome.
- The risk of any STEC-infected patient of developing HUS depends on the toxin type and is relevant for Stx2, negligible for Shiga toxin 1 (Stx1), and inexplicably intermediate when the same amount of Stx2 is associated with Stx1.

**What does this paper add?**

- We have observed that the presence of Stx1 in human blood reduces the number, the size, and the Stx2 content of the extracellular vesicles involved in the pathogenesis of HUS.
- Our findings would help to explain the epidemiological association between STEC producing Stx2 and HUS.
