# Supplementary material for: Shiga Toxin 1a Blunts Shiga Toxin 2a-pathogenic Effects in Blood
Source: Thromb Haemost. 2026 Feb 24;126(11):1133–42. doi: 10.1055/a-2805-9903 (PMC13722531; doi:10.1055/a-2805-9903)
Supplement: Supplementary file 1 [file 10-1055-a-2805-9903_28175520.pdf]

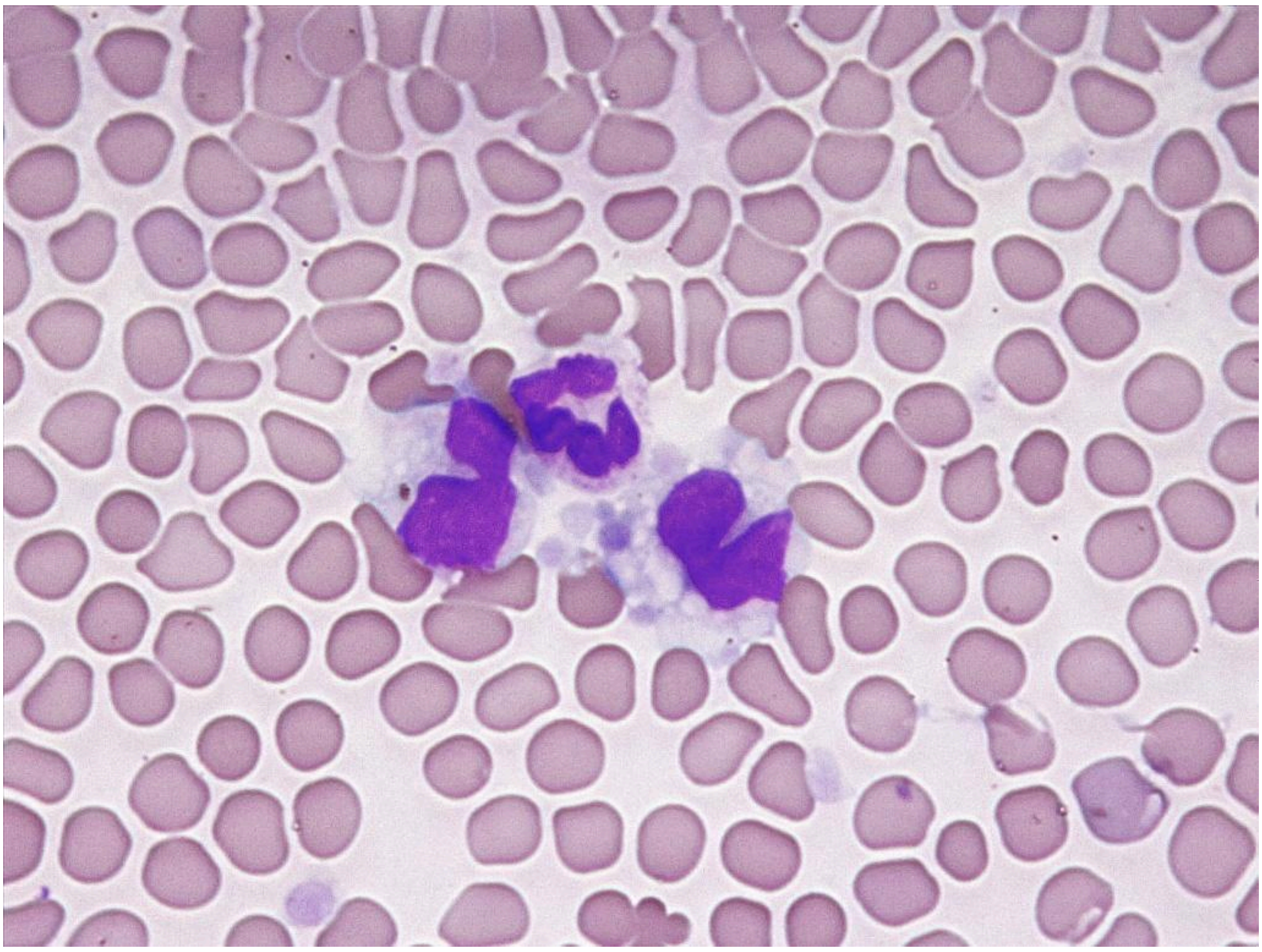

Supplementary Fig. S1 Peripheral blood smear after treatment of blood with Shiga toxin 2. A blood sample from a representative donor was anticoagulated with CPD (citrate-phosphate-dextrose solution) and treated with 2 nM Stx2 as described in Methods. The micrograph shows three leukocytes (one neutrophil and two monocytes) adhering to platelets (May-Grünwald Giemsa stain, 100x magnification).

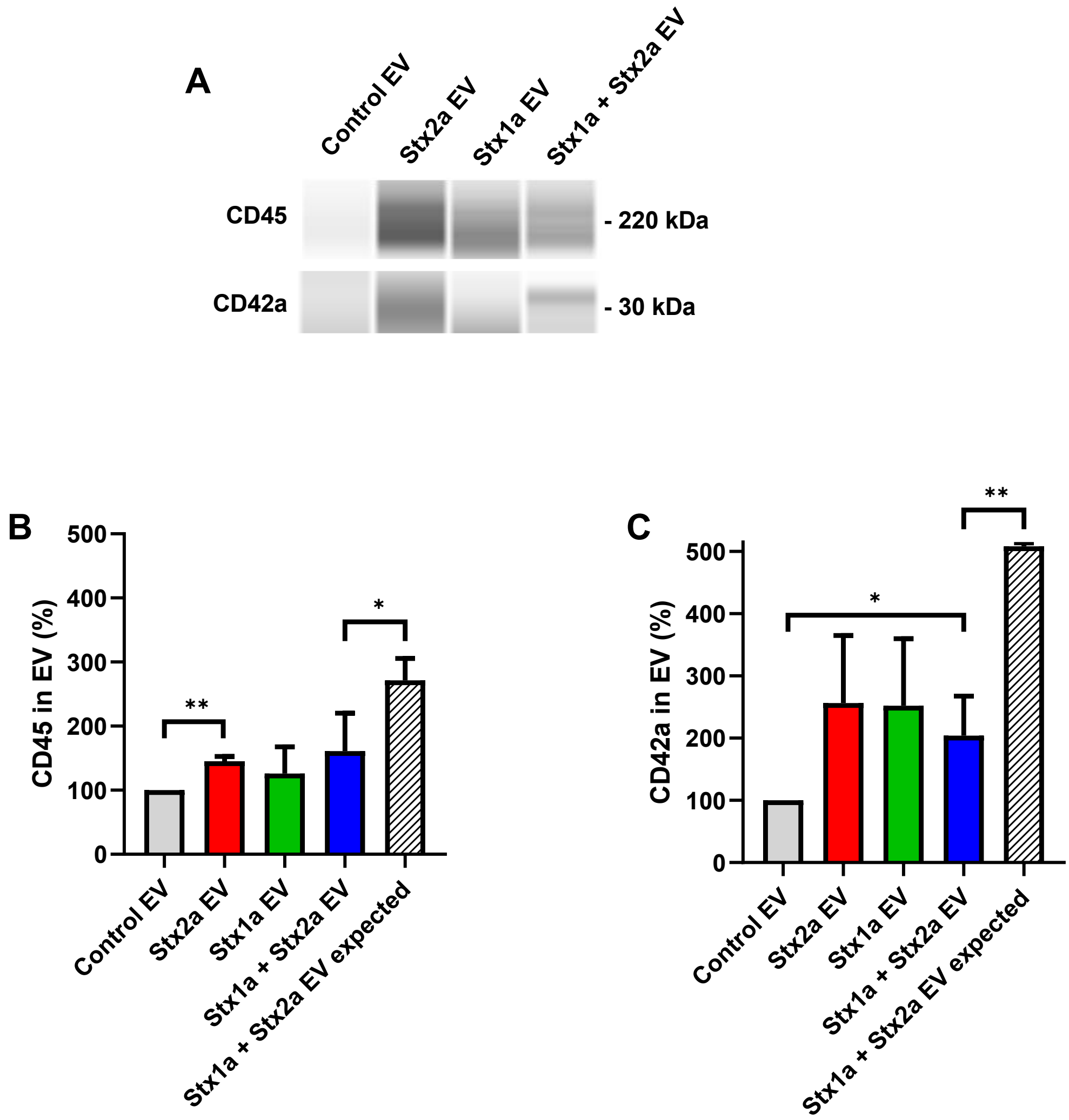

Supplementary Fig. S2 Cellular origin of the EV produced by human blood cells challenged with Stx. A) WES analysis of the leukocyte marker CD45 or of the platelet marker CD42a derived from human EV isolated from a representative donor. B) Data are expressed as mean ± SD (n=3) of the percentage of CD45 determinations with respect to control obtained by analysing the EV isolated from 3 different donors. The expression of CD45 in Stx1a+Stx2a-induced EV was also compared to the sum of the values obtained in Stx1a-induced EV and Stx2a-induced EV. C) Data are expressed as mean ± SD (n=3) of the percentage of CD42a determinations with respect to control obtained by analysing the EV isolated from 3 different donors. The expression of CD42a in Stx1a+Stx2a-induced EV was also compared to the sum of the values obtained in Stx1a-induced EV and Stx2a-induced EV. * $p < 0.05$: ** $p < 0.01$

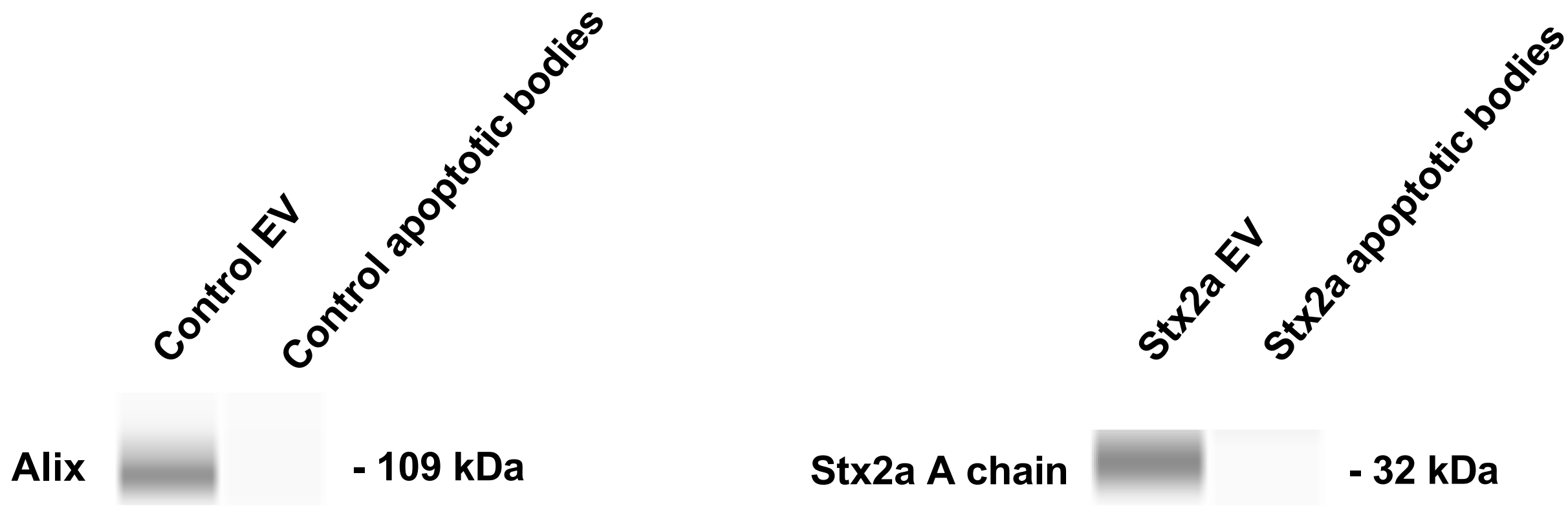

Supplementary Fig. S3 Specific detection of Alix and Stx2a in EV isolated from human blood. Apoptotic bodies (2,600-10,400 g) or large EV (10,400-20,800 g) isolated from human blood treated with PBS or Stx2a, as described in Material and Methods, were compared by WES for the expression of Alix or the association with Stx2a.
